# Honeybee Brain Oscillations Are Generated by Microtubules. The Concept of a Brain Central Oscillator

**DOI:** 10.3389/fnmol.2021.727025

**Published:** 2021-09-29

**Authors:** Brenda C. Gutierrez, Marcelo R. Pita Almenar, Luciano J. Martínez, Manuel Siñeriz Louis, Virginia H. Albarracín, María del Rocío Cantero, Horacio F. Cantiello

**Affiliations:** ^1^Laboratorio de Canales Iónicos, Instituto Multidisciplinario de Salud, Tecnología y Desarrollo (IMSaTeD, CONICET-UNSE), Santiago del Estero, Argentina; ^2^Centro Integral de Microscopía Electrónica (CIME-CONICET-UNT), Tucumán, Argentina

**Keywords:** microtubule, tubulin, honeybee, electrical oscillations, brain, local field potentials

## Abstract

Microtubules (MTs) are important structures of the cytoskeleton in neurons. Mammalian brain MTs act as biomolecular transistors that generate highly synchronous electrical oscillations. However, their role in brain function is largely unknown. To gain insight into the MT electrical oscillatory activity of the brain, we turned to the honeybee (*Apis mellifera*) as a useful model to isolate brains and MTs. The patch clamp technique was applied to MT sheets of purified honeybee brain MTs. High resistance seal patches showed electrical oscillations that linearly depended on the holding potential between ± 200 mV and had an average conductance in the order of ~9 nS. To place these oscillations in the context of the brain, we also explored local field potential (LFP) recordings from the Triton X-permeabilized whole honeybee brain unmasking spontaneous oscillations after but not before tissue permeabilization. Frequency domain spectral analysis of time records indicated at least two major peaks at approximately ~38 Hz and ~93 Hz in both preparations. The present data provide evidence that MT electrical oscillations are a novel signaling mechanism implicated in brain wave activity observed in the insect brain.

## Introduction

Brain waves are coherent patterns of synchronized electrical oscillations thought to represent the activity of large ensembles of active neurons. This evidence is extensively reflected in electroencephalogram (EEG) and local field potential (LFP) recordings (Başar, 1980; Bullock, 1993; Nunez and Srinivasan, 2006). In the mammalian brain, oscillatory electrical activity has been correlated with distinct behavioral states and cognitive tasks (Singer, 1993; Bragin et al., 1995; Fries et al., 1997). However, the oscillatory and synchronized electrical activities in the mammalian brain have also been observed across phyla. LFP recordings have shown similar oscillations after olfactory stimulation in a number of vertebrate and invertebrate brains, including bees, locusts, moths, and flies (Stopfer et al., 1997; MacLeod et al., 1998; Christensen et al., 2003; Tanaka et al., 2009) where synchrony specifies odor recognition at both the cellular (MacLeod et al., 1998) and behavioral levels (Stopfer et al., 1997). Brain LFP recordings of the fruit fly *Drosophila melanogaster* share various key features with physiological correlates in the 40–60 Hz range of visual selective attention in monkeys and humans (Engel and Singer, 2001). Electrical oscillations have been found in systems as disparate as mollusks (Gelperin and Tank, 1990), and rats and mice (Kay, 2003; Schaefer et al., 2006), suggesting a fundamental role in brain computations. Presently, the patterns of electrical activity from organized arrays of neurons cannot be predicted from either the anatomy or histology of the neural substrates or the phenotypic character of neuronal potentials (Bullock, 1993).

Insects recently have become useful models for the study of the relationship between neuronal electrical activity and animal behavior. Insect brains generate oscillatory activity patterns that resemble their vertebrate counterparts. The honeybee in particular has been used to model learning and memory formation and the basis of cognition. While smaller than mammalian brains, the honeybee brain is large both in absolute and relative terms as compared with other insect species. The brain of the honeybee (*Apis mellifera*), containing about 100,000 neurons is about 30–50-times larger than that of the fruit fly (*Drosophila spp*.), where most differences relate to the neural organization of the visual system and mushroom bodies, which are high-order integration centers for sensory inputs (Menzel, 2012). Neural recordings from honeybee brains during learning, memory formation, and retrieval activities have provided evidence for the neural correlates underlying cognitive faculties (Hammer and Menzel, 1995; Okada et al., 2007; Strube-Bloss et al., 2011). Presently, the ultimate molecular fingerprints of the brain oscillations remain to be ascertained.

MTs are unique polymers of the cytoskeleton (Desai and Mitchison, 1997) that form a wide variety of intracellular superstructures (Needleman et al., 2004). In highly polarized cells such as neurons, MTs are required for neuronal growth and maintenance of axons and multiple shorter dendrites (Conde and Cáceres, 2009) that either transmit or receive electrical signals, respectively. MTs are highly charged polymers that behave as biological transistors supporting, amplifying, and axially propagating electrical signals (Priel et al., 2006). Recent studies also demonstrated that assemblies of MTs generate spontaneous, self-sustained, electrical oscillations. This phenomenon was observed in bundles of rat brain MTs (Cantero et al., 2018) that also elicited high synchronized trains of current oscillations that mimicked bursts of action potentials. This electrical activity was richer than that reported for bovine brain MT sheets (Cantero et al., 2016) and more recently observed in isolated MTs (Gutierrez et al., 2020). Thus, the nature of the MT assemblies may be relevant in their nonlinear electrical outcome.

In the present study, we applied the patch clamping technique to MTs obtained from the honeybee brain, and demonstrated the presence of spontaneous electrical oscillations with prominent peaks around 40 Hz and 90 Hz and thus were partially similar to those observed in similar preparations of mammalian MTs (Cantero et al., 2016, 2018). Interestingly, local field currents generated in Triton X-permeabilized, but not the intact whole brain preparation, also displayed spontaneous oscillations with similar frequency spectra. The encompassed data suggests that the cytoskeleton mediates intracellular electrical signals, which may be a central phenomenon of brain tissue.

## Materials and Methods

### Animals

Honeybee (*Apis mellifera*) summer workers were supplied by a professional apiary, located in the agricultural city of La Banda, Santiago del Estero (27°44′S 64°15′W) at 191 m over sea level. Animals were collected between late February and March, transported to the laboratory, and supplied with water *ad libitum* until processing.

### Honeybee Brain MT Preparation

**Figure 1:**
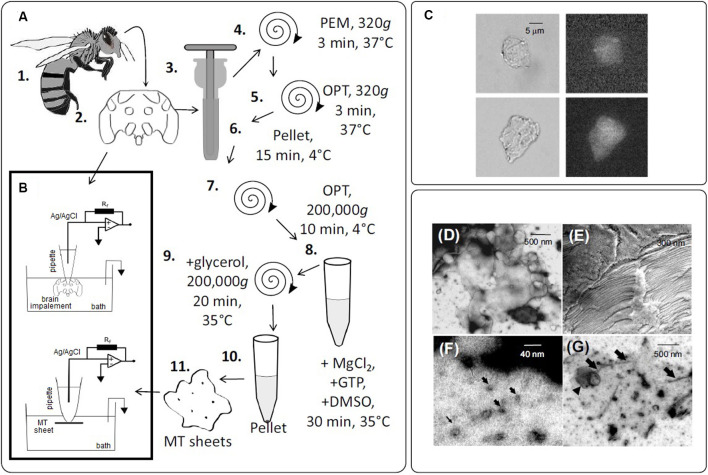
Experimental setup to obtain bee brain microtubules (MTs). **(A)** The sequence of steps in the preparation of honey bee brain MTs. (1.) Worker bees were anesthetized by cold and the brain dissected (2.). Brains were homogenized (3.) and centrifuged (4–10) in several steps. MTs sheets were obtained in the final pellet (11.). **(B)** Schematics of the patch-clamp configuration used to record electrical oscillations from either isolated brain MTs (Top) or MTs sheets (Bottom). **(C)** DIC images of MT sheets (Left), and fluorescent labeling of the same sheets with a FITC-anti-α-tubulin antibody complex (Right) ×40. **(D)** TEM of an MT sheet. **(E)** SEM of stacked MT sheets. **(F,G)** Negative staining of MT sheets. Arrows indicate isolated MTs.

A tubulin-enriched brain cytoplasm preparation from honeybee brains was obtained with a technique adapted from Fourest-Lieuvin (2006) with modifications, as indicated (Figure 1A). Briefly, honeybees were immobilized by exposure to cold (5 min at 4°C) and decapitated. Brain dissection and preparation were conducted as indicated (Carreck et al., 2013), with modifications. The bee head was homogenized for a few seconds in a blender set at low speed in PEM buffer (containing in mM: 100 PIPES, pH 6.7; 1.0 EGTA and 1.0 MgSO_4_) by passes with a Teflon-in-glass homogenizer. Subsequently, centrifugation was performed at 320g for 3 min at 37°C. The pellet obtained was washed with PEM, and a new centrifugation was again performed at 320g for 3 min. The pellet was resuspended for cell lysis in 40 ml of OPT Buffer at 37°C (in mM: 80 PIPES pH 6.7, 1 EGTA, 1 MgCl_2_, and 0.5% Triton-X100, 10% Glycerol, 1 μM pepstatin, 400 μM PMSF). The product of cell lysis was centrifuged at 320g for 3 min at 37°C and the supernatant was carefully discarded. The pellet obtained was resuspended in 2 ml of OPT at 4°C and incubated for 15 min on ice. At the end of this incubation, the sample was ultracentrifuged at 200,000g for 10 min at 4°C. The supernatant, called HOPT extract, was collected. Subsequently, 9 ml of HOPT extracts were supplemented with 5 mM MgCl_2_, 1 mM GTP and 5% DMSO (final concentrations). The solution was then incubated for 30 min at 35°C to allow polymerization of the MTs. The sample of polymerized MTs was placed on a PEM cushion, 60% glycerol and 400 μM PMSF at 35°C, and ultracentrifuged at 200,000g for 20 min at 35°C. The MTs were washed, without resuspension, with 3 ml of PEM50 (35°C) (in mM: 50 PIPES, pH 6.7, 1 EGTA, 1 MgCl_2_, and 1 μM pepstatin and 400 μM PMSF). The pellet was resuspended in a little volume of PEM. Finally, the resulting tubulin suspension was aliquoted and stored at −20°C until use.

### Immunolabeling

Honeybee brain MTs were immunochemically labeled with an anti-α-tubulin antibody raised in rabbit against amino acids 149–448 of human α-tubulin (H-300, sc-5546, Santa Cruz Biotechnology Inc) that was used at 1:100 dilution. The secondary antibody used for tubulin staining was a FITC-tagged bovine anti-rabbit IgG-R (sc-2367, Santa Cruz Biotechnology Inc, CA) used at a 1/100 dilution. Samples were viewed under DIC and fluorescence microscopy with an inverted Olympus IX71 microscope connected to a digital CCD camera C4742-80-12AG (Hamamatsu Photonics KK, Bridgewater, NJ). Images were collected with the IPLab Spectrum (Scanalytics, Viena, VA) acquisition and analysis software, running on a Dell-NEC personal computer.

### Electrophysiology of Triton X-Permeabilized Honeybee Brain

Electrical recordings from intact, *ex vivo*, bee brain were obtained as for the loose-patch-clamp configuration where command voltages (*V_cmd_*) were applied inside the brain matter of the “open skull” exposed tissue. Previously, the tissue was permeabilized for at least 10 min in Triton-X (10%) or 4 h in a KCl solution with Triton-X (1%). Electrode setup was similar to that of patch clamping with pipettes filled with a solution containing, in mM: KCl 140, NaCl 5, EGTA 1.0, and HEPES 10, adjusted to pH 7.18 with KOH. All other details were similar to those from patch clamping experiments. Wherever indicated Paclitaxel (Taxol Equivalent, Invitrogen™, P3456) was prepared as per the manufacturer’s recommendations and added at the indicated concentration.

### Electrophysiology of Honeybee Brain MT Sheets

Approximately 2 μl of the MT sheet preparation was added to the dry surface of the patch clamp chamber, letting it rest for 5 min before adding 400 μl of saline solution. Experiments were conducted under symmetrical conditions, with an “intracellular” Ca^2+^ free bathing and patch pipette solution containing (in mM): KCl 140, NaCl 5, EGTA 1.0, and HEPES 10, adjusted to pH 7.2 with KOH as previously reported (Cantero et al., 2016). Electrical recordings were conducted with a miniaturized patch-clamp amplifier, ePatch, from Elements (Cesena, Italy) with a recording range between ± 200 nA, voltage stimulus range ± 500 mV, and a maximum signal bandwidth of 100 kHz (Figure 1B). Patch pipettes were made from soda lime 1.25 mm internal diameter capillaries (Biocap, Buenos Aires, Argentina) with a tip diameter of ~4 μm and tip resistance in the order of 5–15 MΩ. Voltage clamp protocols only included step-wise holding potentials (gap-free protocol), from zero mV. Electrical signals were acquired and filtered at 10 kHz, digitized with an analog-digital converter (Digidata 1440A, Molecular Devices), and stored in a personal computer with the software suite pCLAMP 10.0 (Molecular Devices), also used for data analysis. Sigmaplot Version 11.0 (Jandel Scientific, Corte Madera, CA) was used for statistical analysis and graphics. Power spectra of unfiltered data were obtained by the Fourier transform subroutine of Clampfit 10.0.

### Electron Microscopy

#### Scanning Electron Microscopy

SEM was conducted at 3 kV with a scanning electron microscope CrossBeam 340, Carl Zeiss (NTS GmbH, Germany, LANAIS-MIE-UBA-CONICET). Briefly, 100 μl MT aliquots were placed onto 10-mm coverslips for electron microscopy and kept at room temperature for 1 h to achieve adhesion. Samples were afterward mounted on aluminum stubs and sputtered with gold using a sputter coater (JEOL model JFC-1100).

#### Negative Staining

Transmission electron microscopy (TEM) was conducted with a Zeiss LIBRA 120 transmission electron microscope (CIME-CONICET-UNT). Briefly, 20 μl of the MT suspension was deposited onto a piece of Parafilm forming a drop, and a 400-mesh nickel grid with a Formvar carbon film was placed over each drop for 5 min. The excess sample was discarded with filter article from the edge of the grid. The samples were then stained for 1 min with 2% aqueous uranyl acetate, removing excess staining from the grids with filter article, and allowed to air dry. The grids were examined immediately afterward.

## Results

### Electrical Activity of Honeybee MTs Sheets

To obtain electrical information from honeybee brain MTs, brain material was obtained, processed, and kept in an Ca^2+^-free “intracellular-like” solution containing high KCl (140 mM) and 1 mM EGTA (see “Materials and Methods” section, Figure 1). DIC and fluorescent microscopy with an anti-α-tubulin antibody proved MT localization in the honeybee brain sheets (Figure 1C). Also, negative staining (NS), a rapid qualitative method for analyzing high resolution structures at the electron microscopy EM level was used to characterize the ultrastructure of the MT sheets (Figures 1D–G). In our case, NS revealed multiple sheets varying in size and form and with heterogeneous structural assemblies of MTs inside (Figure 1D). It is to note however, that NS involves deposition of heavy atom stains such that the flattening and opening up of cylindrical MTs into flat sheets could be observed. Nevertheless, TEM images also showed cross sections of isolated MTs and bundles (Figure 1F) and lacing MT sheets (Figure 1G). In addition, SEM showed stacked sheets in unstained and unfixed material (Figure 1E).

**Figure 2:**
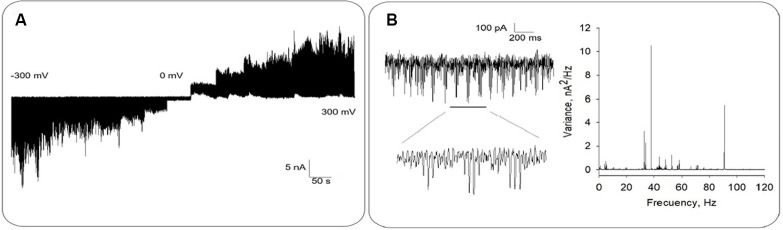
Electrical oscillations from honey bee brain MT sheets. **(A)** Representative electrical recording of an MT sheet at different holding potentials between ±300 mV, in 50 mV voltage steps. **(B)** Left. Expanded tracing shows electrical oscillations at −200 mV. Data representative of *n* = 14 experiments. Right. The panel shows the Fourier spectra of time records (Left) displaying fundamental frequencies at ~38 Hz and ~91 Hz.

**Figure 3:**
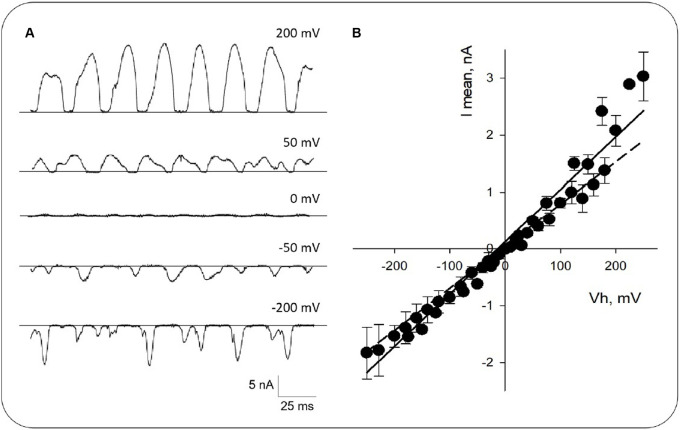
Current-to-voltage curve obtained from honeybee brain MT sheets. **(A)** Expanded tracings at different voltages as indicated. **(B)** Mean current-to-voltage relationship obtained in symmetrical KCl. Experimental data (Black symbols) are shown as Mean ± SEM of *n* = 14 experiments. The solid line represents the linear fitting of the data with a mean conductance of ~9 nS. The dashed line represents linear fitting from −250 mV to −150 mV.

Bee brain MT sheets (*n* = 70) were electrically recorded with a patch clamp amplifier as previously reported (Cantero et al., 2016, 2018). The tip resistance of the patch pipette was 10.2 ± 1.20 MΩ (*n* = 14) under symmetrical saline conditions. Apposition of the pipette tip onto an MT sheet increased the resistance to 214 ± 36 MΩ (*n* = 14), in contrast to higher seal resistances usually obtained with mammalian MT sheets (Cantero et al., 2016, 2019). The difference in sealing electrical resistance of the MT sheets may implicate the availability of either ionic species associated with their formation (Wolf et al., 1993), and/or yet undetermined interacting proteins in the preparation. Approximately 96% (67/70) voltage-clamped MTs displayed spontaneous, self-sustained electrical oscillations (Figures 2A, 3A) that responded directly to the magnitude and polarity of the electrical stimulus. Fourier spectra showed very distinct peaks, being the most prominent at two fundamental frequencies, ~38 Hz and ~93 Hz (Figure 2B).

The amplitude and the oscillatory pattern depended on the applied voltage (Figure 3A). The current-to-voltage relationship was highly linear between −250 and 150 mV (Figure 3B, Dashed line), with outward rectification at higher positive potentials, an experimental positive conductance of 18.1 ± 4.8 nS (+150 mV to +250 mV), and a negative conductance of 5.3 ± 1.2 nS (−250 mV to −150 mV; *n* = 14, *p* < 0.05, Figure 3B), respectively. The average linear fitting had a mean conductance of 9.2 ± 0.3 nS (*n* = 14, Figure 3B, Solid line).

**Figure 4:**
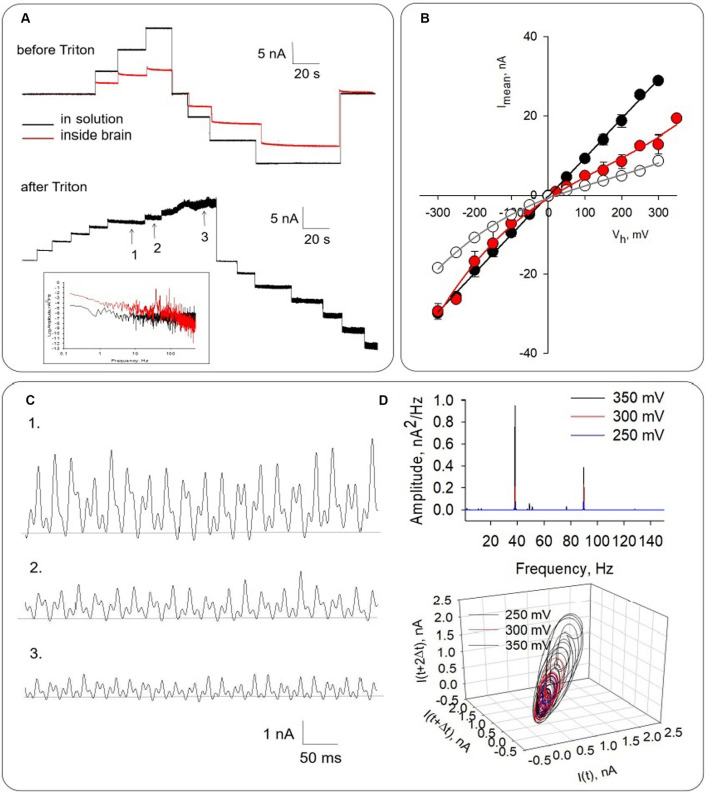
Honeybee brain electrical activity before and after Triton-X permeabilization. **(A)** Top. Electrical response observed after brain impalement. The black line indicates the response of the pipette tip in solution. The red line tracings show the electrical current inside the brain mass in the absence of Triton-X. Bottom. Electrical response observed approximately 30 min after the addition of Triton-X. Numbers indicate regions that are expanded in **(C)**. **(B)** Mean current-to-voltage relationship obtained in symmetrical KCl for pipette in the solution (Black symbols), after impalement either in the absence (Open symbols) or presence of Triton-X (Red symbols). The curves were well approximated by polynomial functions (solid lines). Symbols represent mean ± SEM for five (filled symbols), three (open symbols), and four (red symbols) experiments, respectively. Numbers 1–3 indicate the expanded regions shown in **(A)**. **(C)** Electrical oscillations of local field currents from Triton-X permeabilized honeybee brain. Panels shows from Top to Bottom, electrical recordings at 350, 300, and 250 mV driving voltage, respectively. **(D)** Top. Power spectrum shows fundamental frequencies at ~38 Hz and ~93 Hz, which did not change with changes in the driving force. Bottom. Three-dimensional phase-space portraits showing limit cycles. Delay time for first and second derivatives adopted for phase portraits was 10 ms.

### Electrical Activity of Permeabilized Honeybee Brain

To explore the oscillatory behavior of MTs on honeybee brain function, a different set of experiments was conducted by measuring directly local field currents from the intact brain (Figure 4). For these studies, the whole honeybee brain was dissected and incubated in the “intracellular-type” high KCl saline solution to depolarize the brain tissue. The voltage-clamp patch pipette was then used to assess LFP in the form of electric currents at the location of the pipette in symmetrical ionic conditions. The tip conductance was first measured in saline solution, which was highly linear as shown (Figures 4A,B, black symbols). Insertion of the pipette in the brain mass (Figure 4A, Top) was associated with an inward rectification of the current-to-voltage relationship (Figure 4B, Open symbols). Under “free-floating” conditions with zero mV applied voltage, the tip resistance increased from 16.8 ± 2.2 MΩ to 69.9 ± 18.5 MΩ (*n* = 9) after insertion in the brain mass, and an average current of 28.0 ± 2.52 pA (*n* = 7). To further explore whether intracellular (cytoskeleton-associated) oscillations occurred, an aliquot of a 10% Triton-X solution was added to the chamber to induce membrane permeabilization (Figure 4A, Bottom). Expansion of electrical recordings showed that the amplitude of the oscillations increased with the holding potential (Figure 4C), and an increase in conductance (Figure 4B, Red symbols). The power spectrum of these oscillations (Figure 4D, Top) showed clearly identifiable principal frequency peaks at ~38 Hz and ~93 Hz, which remained constant with changes in the driving force. Three-dimensional phase-space portraits showed limit cycles that increased with the applied voltage (Figure 4D, Bottom) and disclosed changes in dynamics of the oscillations.

**Figure 5:**
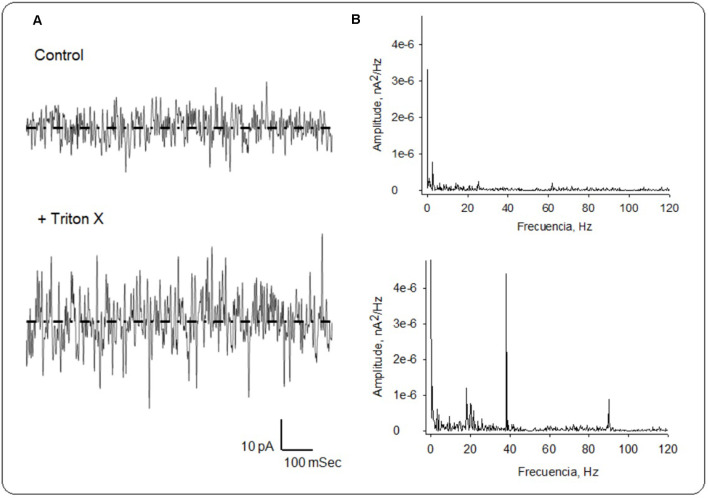
Local field currents in the honeybee brain. **(A)** Currents were collected and averaged (*n* = 3) for spontaneous recordings at zero mV before (Top), and after the addition of Triton X (Bottom). **(B)** Fourier spectra of recordings on Left were obtained after notch (50 and 100 Hz), and Bessel (120 Hz) filtering. Dashed lines represent average currents at zero mV of 28 pA and 55.7 pA, for the control and Triton X-treated conditions, respectively.

Within 30 min of incubation in Triton-X at room temperature currents increased to 55.7 ± 8.45 pA (*n* = 3), thus statistically doubling the control value (*p* < 0.05, Figure 5A). The cytosolic increase in current represented a very robust LFP at the pipette tip in the order of 831 μV. This increase in conductance was associated with the onset of electrical oscillations (Figure 5). The power spectrum of these oscillations (Figure 5B) showed at least two principal frequencies at ~38 Hz and ~93 Hz, as well as a wider peak in the 18–22 Hz range. The oscillatory currents from the permeabilized brain were made more evident and responded to the magnitude and polarity of the applied voltage. No attempt was made to identify specific brain regions in the preparation. However, at least one experiment in which the mushroom body was impaled showed strong oscillatory behavior (data not shown).

**Figure 6:**
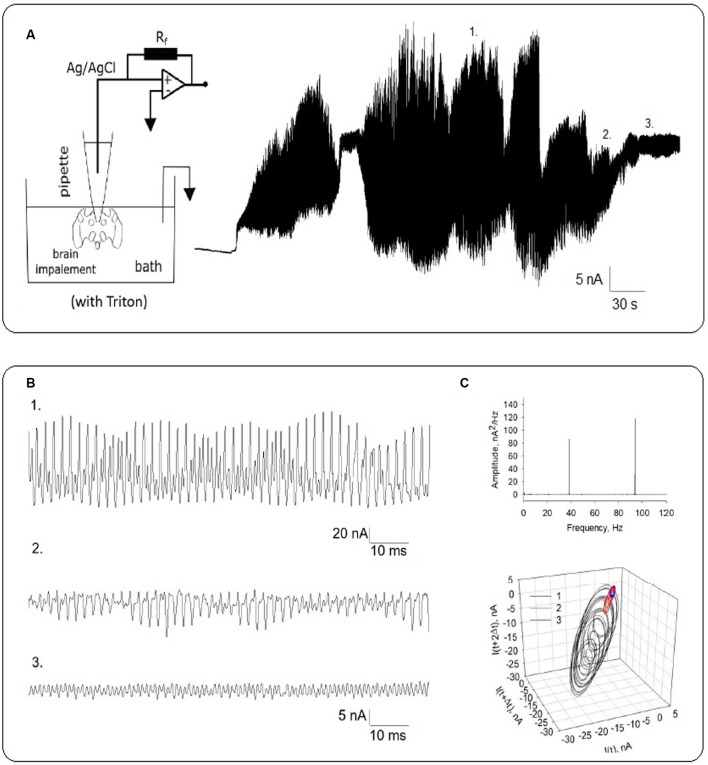
Local field oscillatory currents from Triton-X permeabilized honeybee brain. **(A)** Rich electrical oscillatory patterns were observed after higher voltages (−400 mV) were applied. The electrical setup is schematized on the Left. **(B)** Expanded recordings show different patterns of electrical oscillations at the same voltage. Numbers 1 through 3 indicate the expanded region indicated in **(A)**. **(C)** Top. The power spectrum shows fundamental frequencies at ~38 Hz and ~93 Hz. Bottom. Three-dimensional phase-space portraits showing limit cycles. Delay time for first and second derivatives adopted for phase portraits was 10 ms.

LFPs changed dramatically even in the absence of changes in driving force (Figure 6A), suggesting a complicated dynamic behavior. Expanded regions show different patterns of electrical oscillations observed at the same voltage (Figure 6B). The power spectrum also shows fundamental frequencies at ~38 Hz and ~93 Hz (Figure 6C, Top). Three-dimensional phase-space portraits showed limit cycles, evidencing the change in the oscillatory behavior without changes in the driving force (Figure 6C, Bottom).

**Figure 7:**
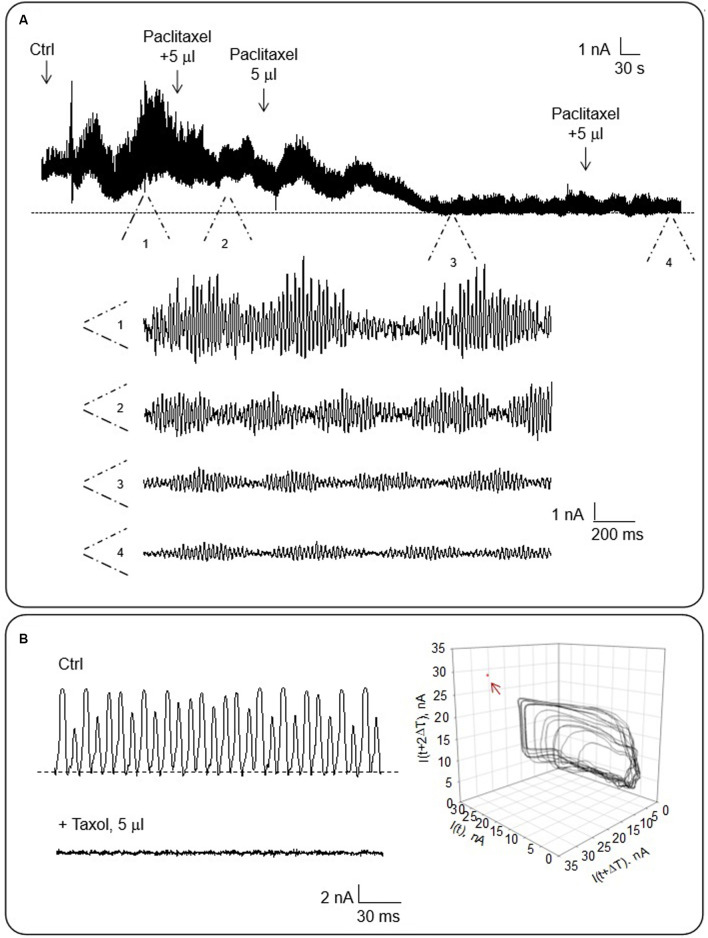
Effect of Paclitaxel on the electrical oscillations of the honeybee brain. The effect of taxol on the electrical current oscillations observed at a holding potential of 200 mV in symmetrical KCl. **(A)** Top. The panel shows time series of oscillatory currents before and after the addition of various concentrations (as indicated) of Paclitaxel to the bath. Bottom. Expanded regions (indicated as 1–4) of the oscillatory currents shown in Top (*n* = 3). **(B)** Left. The panel shows representative electrical recordings of an MT sheet before and after Paclitaxel addition. Right. Three-dimensional phase-space portraits showing monoperiodic limit cycles. Delay time (T) for first and second derivatives adopted for phase portraits was 10 ms. Arrow points to the absence of oscillations in the presence of Paclitaxel.

To evaluate the contribution of MTs to the honeybee brain electrical oscillations, we assessed the effect of the MT stabilizer Paclitaxel that eliminates the electrical oscillations of other MT preparations (Cantero et al., 2016, 2018). The drug was added after incubation in Triton-X of the whole brain. The spontaneous electrical oscillations were significantly reduced after subsequent additions of the drug as shown in Figure 7A (*n* = 3) to reach complete inhibition. Moreover, the electrical oscillations were also completely inhibited when Paclitaxel was added to electrically active MT sheets (Figure 7B, *n* = 3). These results enforce the idea that MTs are fundamental participants of the intracellular oscillations observed in the honeybee brain.

## Discussion

The present study provides the first direct evidence that the honeybee brain produces intrinsic electrical oscillations that are mediated by intracellular MTs. The oscillatory LFP in the permeabilized whole brain preparation was observed under “intracellular-like” bathing conditions only after tissue permeabilization with Triton-X. Confirmation that the electrical oscillations of the honeybee brain were generated by MTs was obtained by patch clamping of honeybee brain 2D MT sheets that showed similar patterns and power spectral densities. In both cases, the oscillatory behavior peaked at frequencies around 40 Hz and 90 Hz. The findings are in general agreement with the oscillatory behavior of mammalian brain MT sheets (Cantero et al., 2016, 2020) and membrane-permeabilized murine hippocampal neurons (Cantero et al., 2018). A couple of differences are worth noting, however. First, a prominent 93 Hz band was usually observed that was unapparent in recordings from cow and rat brains. Another interesting finding was a wider range peak around 18–22 Hz that was only observed in the absence of voltage stimulation. Thus, the honeybee brain held somewhat richer electrodynamic properties as compared to those observed in mammalian MTs (Cantero et al., 2016, 2018), but suggesting a similar general phenomenon of brain tissue. Interestingly, recent studies in isolated brain MTs showed the richest pattern of frequencies (Gutierrez et al., 2020), suggesting a phenomenon of synchronization and coherence in MT assemblage in different structures.

Synchronized LFP oscillations in the brain are thought to represent a coherent activity of large assemblies of neurons. Although mammalian brain electrical oscillations have been correlated with the execution of complex cognitive tasks (Singer, 1993; Bragin et al., 1995; Fries et al., 1997), “brain waves” are widely found throughout phyla from lower invertebrates to mammals (Adrian and Matthews, 1934; Bullock and Başar, 1988; Bullock, 1993), and biological systems as disparate as mollusks (Gelperin and Tank, 1990; Schütt and Başar, 1992), moths (Christensen et al., 2003), locusts (Laurent and Naraghi, 1994), rats, mice and monkeys (Kay, 2003; Schaefer et al., 2006; Ray and Maunsell, 2011). This widespread electrical activity may not be explained by differences in brain anatomy (Bullock, 1993), thus posing the possibility of the existence of an underlying universal mechanism for the genesis of electrical oscillations in the brain (Bullock and Başar, 1988).

Both spontaneous and event-related brain oscillations have been observed and categorized into five frequency bands: delta (0.5–3.5 Hz), theta (4–7 Hz), alpha (8–12 Hz), beta (13–30 Hz), and gamma (>30 Hz) that can be roughly separated into two groups. Lower frequency, delta, theta, and alpha oscillations encase global processing modes that span relatively large cortical regions, while higher frequency waves include the beta and gamma ranges, which are usually distributed over more limited topographic areas (Nunez and Srinivasan, 2006). Slower oscillations are linked to more basic and general classes of processes. Delta oscillations that dominate the EEG of waking reptiles and lower vertebrates such as reptiles, amphibians, and fish are associated with evolutionarily old basic processes. In humans, these waves are prominent only in the early developmental stages of development, and during slow-wave sleep. Delta oscillations increase during hunger, sexual arousal, and are disrupted in alcohol and substance users (reviewed in Knyazev, 2012). Theta oscillations dominate in lower mammals, while alpha oscillations are associated with more advanced systems such as adult humans (Knyazev and Slobodskaya, 2003). Alpha oscillations around 10 Hz are the strongest rhythm (Berger, 1929), measurable from the human scalp EEG that is related to cognitive phenomena and it has been linked to cognitive functions such as attention and memory in humans and other vertebrates (Palva and Palva, 2007; Jensen and Mazaheri, 2010). A recent study by Popov and Szyszka (2020) found prominent, olfactory-regulated, mushroom body-associated spontaneous 18 Hz oscillations in the honeybee brain that exhibited properties of alpha oscillations in humans and non-human primates. However, odor-stimulated changes in the power spectrum are accompanied by an increase in the low gamma band (20–40 Hz oscillation) and above 40 Hz high gamma activity (Buzsáki and Silva, 2012). The fruit fly’s LFP responses in the 40–60 Hz range share several key features of visual selective attention in monkeys and humans. Insect brains generate oscillatory activity patterns that may be associated with our findings, including spontaneous 10–20 Hz oscillations in water beetles (Adrian and Matthews, 1934) and honeybees (Ritz et al., 2001), sleep state-dependent 10 Hz oscillations in fruit flies (Yap et al., 2017), odor-induced 20–100 Hz oscillations in locusts (Laurent and Naraghi, 1994), moths (Heinbockel et al., 1998) and bees (Stopfer et al., 1997; Okada et al., 2007; Denker et al., 2010) and visual stimulus-induced 20–30 oscillations in fruit flies (van Swinderen and Greenspan, 2003; van Swinderen et al., 2009). In *Drosophila* exposure to visual objects modulated 20–30 Hz oscillatory activity, which requires the output of neurons from the mushroom body (van Swinderen et al., 2009). LFP oscillatory activity in flies recorded centrally in the brain is similar to that recorded in mammals (Nitz et al., 2002; van Swinderen and Greenspan, 2003), and olfactory stimulation instead increased LFPs in the 70–80 Hz range (van Swinderen and Greenspan, 2003). The recorded oscillatory LFP in the present study is consistent with robust gamma range, both lower (~30–40 Hz) and higher (70–90 Hz) frequencies, which are believed to play a role in cognition. Accordingly, aberrant gamma oscillations are associated with cognitive disorders, including Alzheimer’s disease and Fragile X syndrome (Mably and Colgin, 2018). The present experimental model may offer new insights into the correlations between brain waves and brain cognitive function.

Although the origin of honeybee’s brain oscillations has not yet been determined, odor-induced 30 Hz oscillations were observed in the input region of the mushroom body that may reflect oscillatory spike synchronization across presynaptic neurons (Laurent and Naraghi, 1994). Event-related gamma oscillations are the most prominent oscillatory response in the frequency range of 40–60 Hz in the cat visual cortex (Eckhorn et al., 1988; Gray and Singer, 1989). Widespread findings of this frequency range have been linked to both sensory and cognitive gamma responses. In fact, oscillations observed in mammalian MTs (Cantero et al., 2016, 2018) are rather similar to the current sources of the visual cortex in lower vertebrates (Prechtl et al., 2000).

For their size, brains are the most complex systems known (Allman, 1999). Thus, following the premise that learning creates physical memories traces as changes in neural activity and communication, it is appealing to pose the hypothesis that for neural and cellular correlates of learning and memory, the electrical behavior of intracellular MTs may provide a general oscillator mechanism to drive their function and synchronization. Given that all organisms share a common ancestry, some of the most basic features of brains are likely to be found in common intracellular structures. Evolutionarily speaking highly conserved cytoskeletal structures that originally provided means of motility may have become the core of sensory functions. Microtubule-based organelles provide an interesting link between the cytoskeletal polymers and sensory functions. Cilia and flagella are fundamental organelles composed of complex structures of MTs, which would imply an evolutionary development of the electrical oscillatory capacity that would be at the center of their universal sensory function and their function as computational devices (Vissol-Gaudin et al., 2021). Interestingly, preliminary results from our laboratory showed that assemblies of the bacterial tubulin ancestor FtsZ also elicit electrical oscillations similar to those observed in mammalian MTs (Bonacina et al., 2020).

In summary, the present study shows sizeable MT-mediated electrical oscillations that pervade the brain of the honeybee and suggests the existence of a central oscillator that could be implicated in the genesis of brain waves. Future experiments will be able to determine whether identifiable regions of the brain may have weaker or stronger oscillatory signals that could also be suitably tested in cognition and behavioral studies. The study provides evidence in support of MTs as a brain central oscillator underlying brain waves.

## Data Availability Statement

The raw data supporting the conclusions of this article will be made available by the authors, without undue reservation.

## Author Contributions

BG carried out experimental procedures. MP provided honeybees and carried out experimental procedure. BG and MC conducted the analysis of the experimental data, and prepared the Figures. HC and MC designed all the electrophysiological experiments. VA designed and analyzed all the EM experiments. LM and MS conducted the SEM and TEM experiments, respectively. BG, MC, and HC wrote the main manuscript text. All authors reviewed the manuscript. All authors contributed to the article and approved the submitted version.

## Conflict of Interest

The authors declare that the research was conducted in the absence of any commercial or financial relationships that could be construed as a potential conflict of interest.

## Publisher’s Note

All claims expressed in this article are solely those of the authors and do not necessarily represent those of their affiliated organizations, or those of the publisher, the editors and the reviewers. Any product that may be evaluated in this article, or claim that may be made by its manufacturer, is not guaranteed or endorsed by the publisher.
